# Physiology

**Published:** 1879-03

**Authors:** 


### PHYSIOLOGY

GOING down Broadway any day, scores of men and women may be observed as unconscious of the presence of their fellow-men, despite the pressing along of the ceaseless crowd, the rattling of wheels, and the din of business, as if they were in the midst of Sahara or some boundless prairie of the West, or in an Indian canoe in mid ocean. There may be noticed at any hour, the compressed lip, the muttering speech, the sharp exclamation, the impatient gesture, and the smothered curse. The public pulse beats fast and high and hard; the machinery of life is running at a rate so abnormal in its rapidity, that it must wear out long before its time, or be shivered to atoms by the unnatural tension.

